# A Large Mass Mimicking Calcified Hematoma of the Skull Showing Involvement of Myeloma Cells and a Good Response to Irradiation

**DOI:** 10.1055/s-0039-1697636

**Published:** 2019-10-18

**Authors:** Takamitsu Fujimaki, Sachiko Hirata, Naruhiko Terano, Kenji Wakiya, Masahito Kobayashi

**Affiliations:** 1 Department of Neurosurgery, Saitama Medical University, Moroyama, Japan

**Keywords:** myeloma, skull mass, calcified hematoma

## Abstract

Patients with multiple myeloma often show skull bone involvement, although in most cases this is manifested as skull erosion and large masses develop only rarely. Here we report a patient who presented with a large cranial mass mimicking a subdural hematoma with calcification. The tumor shrunk with 37.5 Gy of focal irradiation in 15 fractions after biopsy. After irradiation the patient was treated with Bortezomib but died because of adverse events. The differential diagnosis of lenticular lesion of the skull and treatment strategy for large skull mass with myeloma cells are discussed.

Patients with multiple myeloma often show skull bone involvement, although in most cases this is manifested as skull erosion, 1 and large masses develop only rarely. 2 Here we report a patient who presented with a large cranial mass mimicking a subdural hematoma with calcification. After biopsy, the local tumor was treated successfully by irradiation. The diagnostic procedures and treatment strategies for skull tumors, especially large plasmacytomas or myelomas, are discussed.

## Case Report

**Fig. 1:**
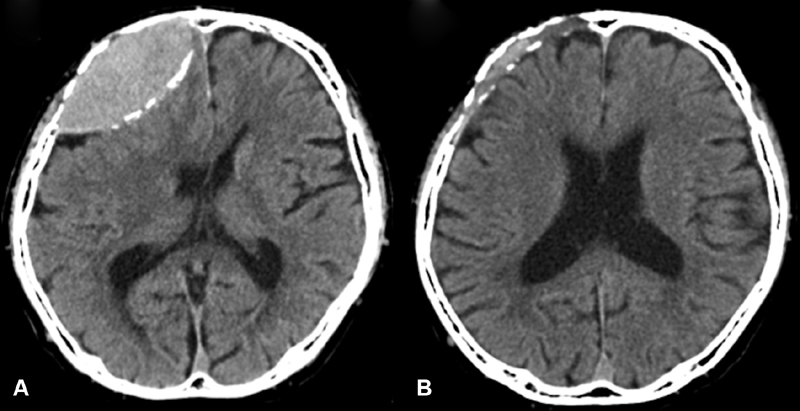
Computed tomography scan on admission ( **A** ). The mass is lenticular with marginal calcification. The tumor disappeared almost completely after 37.5 Gy of irradiation ( **B** ).

**Fig. 2:**
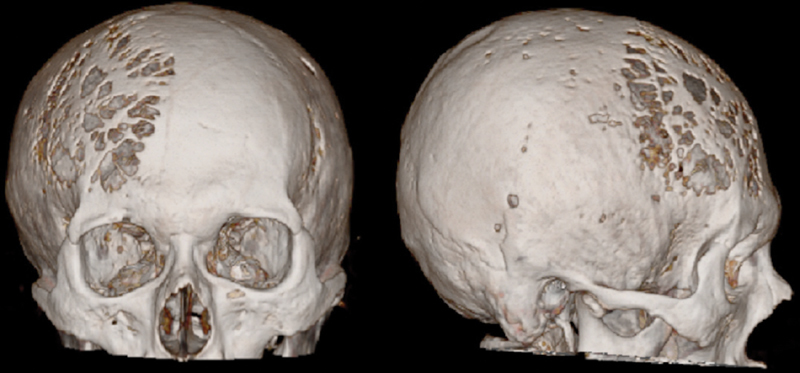
Three-dimensional reconstruction based on the computed tomography scan through a bone window on admission, showing multiple punched-out lesions of the skull.

**Fig. 3:**
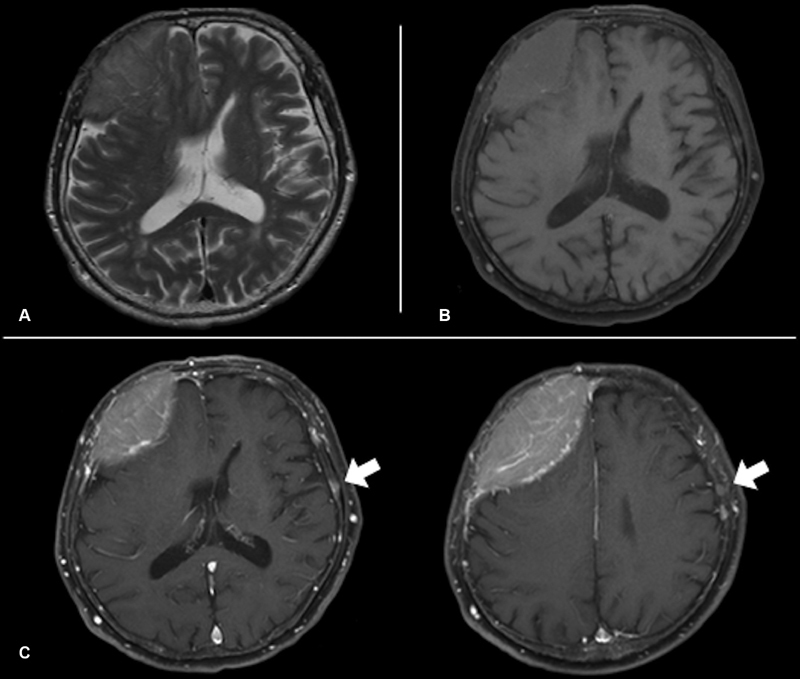
Magnetic resonance imaging on admission. The mass showed iso-signal intensity in T1 ( **A** ), T2 ( **B** ), and diffusion-weighted images ( **C** ). The main mass was well enhanced by gadolinium ( **C** ), and multiple enhanced lesions were observed other than the main mass (arrows).

**Fig. 4:**
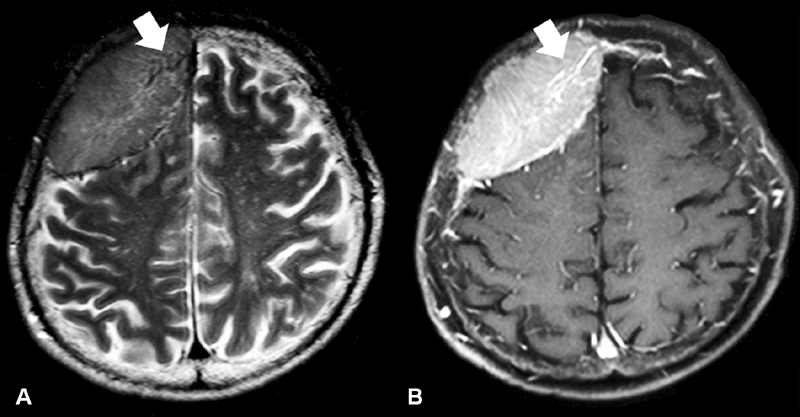
Magnetic resonance imaging on admission showing the vasculature of the tumor. There was a flow void sign on the T2-weighted image ( **A** ) and this part was well enhanced with gadolinium ( **B** ).

**Fig. 5:**
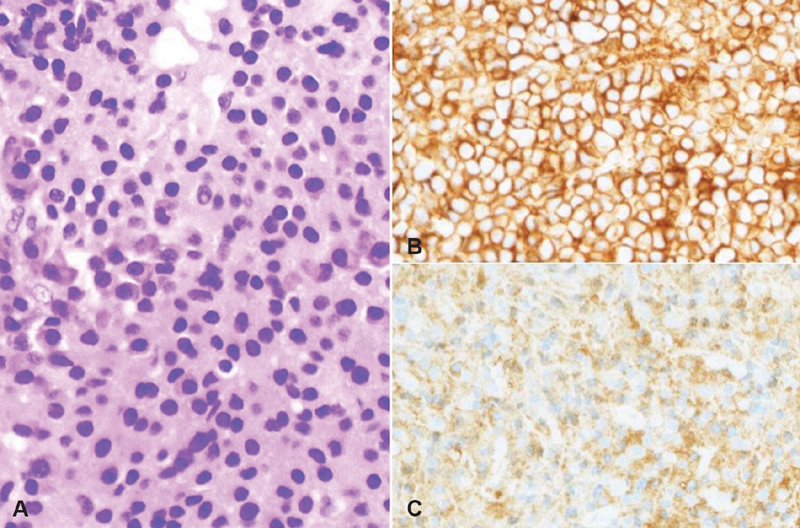
Histopathology of the biopsy specimen, showing massive proliferation of plasmacytoid cells ( **A** ), and immunohistochemistry showing positive staining for CD138 ( **B** ) and immunoglobulin lambda chain ( **C** ).

A 75-year-old man was referred to our Saitama Medical University from a local hospital, where he had presented with gait disturbance and a computed tomography (CT) scan had demonstrated an extra-axial mass, suspected to be a calcified subdural or epidural hematoma. Three months previously, he had suffered a fall in the bathroom without any head trauma, and also had a history of knee joint inflammation. On admission, the findings of detailed neurological examinations were essentially normal, but the patient was unable to walk because of knee pain. A CT scan demonstrated a right frontal mass that compressed the frontal lobe, and calcifications at the border between the mass and the cortex ( Fig. 1 ). There were erosions of the skull overlying the mass, as well as elsewhere on the cranial vault ( Fig. 2 ). Myeloma with skull involvement was strongly suspected. Initial laboratory examinations revealed elevated serum levels of immunoglobulin G (4435 mg/dL) and immunoglobulin lambda chain (292.0 mg/L). Urinalysis was positive for Bence Jones protein (BJP). The tumor was well enhanced by gadolinium contrast, and a flow void with linear enhancement suggestive of large vessels within the tumor was demonstrated by magnetic resonance imaging (MRI) on admission. Some small skull lesions were also present ( Figs. 3 and 4 ). Based on a preoperative diagnosis of myeloma skull invasion, biopsy of the tumor was performed in the area of the cortical bone defect. The tumor was hemorrhagic, but the bleeding was well controlled by bipolar coagulation and compression by cottonoid, since the surgery involved a limited field. Pathological examination of frozen and permanent sections confirmed the diagnosis of myeloma cell invasion ( Fig. 5 ). Local irradiation was started, and the patient was transferred to the Department to Hematology. After 37.5 Gy of irradiation in 15 fractions, the tumor showed marked shrinkage. During the irradiation, dexamethasone (20 mg × 4 days) was also administered. A systemic bone survey revealed erosion of the clavicle accompanied by pain, and therefore local irradiation of this lesion was also performed. After irradiation, bortezomib chemotherapy was started, but this led to disseminated intravascular coagulation and pneumonia as adverse events. Despite intensive treatment, the patient died 70 days after biopsy of the skull lesion. Autopsy was not performed.

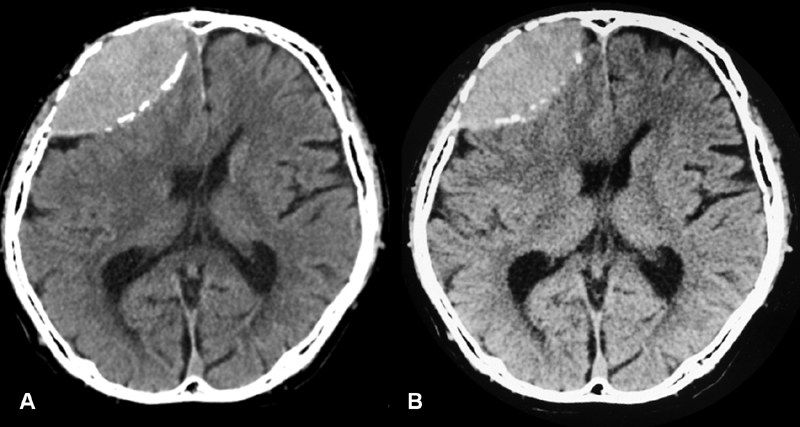



## Discussion

Intracranial lenticular or lens-shaped masses are not rare in the field of neurosurgery. Although epidural or subdural hematomas are the most frequent lesions, lenticular masses with calcification are relatively unusual. According to a PubMed search (113 papers, as of February 6, 2019), calcified subdural hematoma is the most frequent type. About 0.3 to 2.7% of chronic subdural hematomas have calcification. 3 Calcified epidural hematoma and calcified empyema are among the other entities that need to be considered in the differential diagnosis of calcified epidural hematoma. However, a previous review 4 did not include plasmacytoma or a tumor mass composed of myeloma cells. The skull is one of the most frequent sites for myeloma cell invasion, and punched-out or lytic lesions are common. However, presentation in the form of a large mass is quite unusual, 1 2 5 and only a limited number of such cases have been reported. 6 7 Although the present patient was referred to us with a tentative diagnosis of calcified hematoma, we suspected skull involvement of myeloma because multiple lytic skull lesions were present as well as the large mass. This prompted us to perform laboratory examinations for specific markers such as serum immunoglobulin and urinary BJP, to investigate the possibility of myeloma.

With regard to treatment, solitary plasmacytoma can be cured by surgery alone, but adjuvant therapy is mandatory for patients with multiple lesions. The tumors are rather hemorrhagic, and postoperative hemorrhagic complications have been reported. 5 Here, as MRI showed flow void signs and well-enhanced linear features within the tumor, suggestive of high-flow blood vessels, extensive surgical removal was considered to be risky. Biopsy confirmed the diagnosis of cranial involvement. The mass was almost completely resolved after local brain irradiation. Although the patient later died due to complications of chemotherapy after irradiation, our experience indicated that massive skull plasmacytoma can be well controlled with radiation therapy following biopsy to confirm the histology.

## Conclusion

Lenticular lesions of the skull revealed by CT are not always hematomas, and skull involvement of plasmacytoma or myeloma should be borne in mind when the mass is accompanied by multiple lytic lesions. Biopsy followed by irradiation is able to control large invasive skull masses composed of myeloma cells.
